# Effect of thallus melanisation on the sensitivity of lichens to heat stress

**DOI:** 10.1038/s41598-023-32215-1

**Published:** 2023-03-28

**Authors:** Karolina Chowaniec, Ewa Latkowska, Kaja Skubała

**Affiliations:** 1 Institute of Botany, Faculty of Biology, Jagiellonian University, Gronostajowa 3, 30-387 Kraków, Poland; 2 Doctoral School of Exact and Natural Sciences, Jagiellonian University in Kraków, Prof. S. Łojasiewicza 11, 30-348 Kraków, Poland; 3 Laboratory of Metabolomics, Faculty of Biochemistry, Biophysics and Biotechnology, Jagiellonian University, Gronostajowa 7, 30-387 Kraków, Poland

**Keywords:** Plant physiology, Plant stress responses, Ecophysiology

## Abstract

Extreme climatic phenomena such as heat waves, heavy rainfall and prolonged droughts are one of the main problems associated with ongoing climate change. The global increase in extreme rainfalls associated with summer heatwaves are projected to increase in amplitude and frequency in the near future. However, the consequences of such extreme events on lichens are largely unknown. The aim was to determine the effect of heat stress on the physiology of lichen *Cetraria aculeata* in a metabolically active state and to verify whether strongly melanised thalli are more resistant than poorly melanised thalli. In the present study, melanin was extracted from *C. aculeata* for the first time. Our study showed that the critical temperature for metabolism is around 35 °C. Both symbiotic partners responded to heat stress, manifested by the decreased maximum quantum yield of PSII photochemistry, high level of cell membrane damage, increased membrane lipid peroxidation and decreased dehydrogenase activity. Highly melanised thalli were more sensitive to heat stress, which excludes the role of melanins as compounds protecting against heat stress. Therefore, mycobiont melanisation imposes a trade-off between protection against UV and avoidance of damage caused by high temperature. It can be concluded that heavy rainfall during high temperatures may significantly deteriorate the physiological condition of melanised thalli. However, the level of membrane lipid peroxidation in melanised thalli decreased over time after exposure, suggesting greater efficiency of antioxidant defence mechanisms. Given the ongoing climate changes, many lichen species may require a great deal of plasticity to maintain their physiological state at a level that ensures their survival.

## Introduction

Lichens constitute symbiotic self-sustaining partnerships of a fungus and a photosynthetic partner, typically an alga and/or cyanobacterium associated with diverse microorganisms^1^. In these associations, both main symbiotic partners form a structural and functional unit; however, they consist of two organisms of completely different metabolism, and the details of their interactions are still poorly investigated. Lichens can colonise environments hostile to life and certain extreme ecological niches^2^. Many lichen species occur in extremely arid environments, such as deserts or arctic/alpine ecosystems, where they are exposed to high solar radiation. However, they have a remarkable ability to cope with such extreme conditions by several structural and functional adaptations, e.g. the specific structure of the cortex layer^3^, desiccation tolerance^4^, ability to anhydrobiosis^5^, and production of enzymatic and non-enzymatic antioxidants scavenging ROS^6^ or UV-screening secondary metabolites and melanin pigments^7,8^.

Melanism is defined as the occurrence of mostly or entirely dark-pigmented individuals, either as polymorphisms within the species or as established differences among closely-related species^9^. Melanins are brown or black high molecular weight pigments present in almost all biological groups^10^, produced by oxidative polymerisation processes of phenolic and indolic compounds^11^. Melanin synthesis is one of the most universal and, simultaneously, the most enigmatic adaptation of living organisms to variable conditions on Earth. Melanisation is common in organisms exposed to extreme environmental conditions and live in hot and cold deserts, high mountains or areas contaminated with toxic trace elements and ionising radiation^12^. Moreover, melanin production in fungi could be induced in response to environmental stress, such as exposure to UV, toxic compounds, desiccation, hyperosmotic shock, extreme temperatures or pH or a limited pool of nutrients^11,13^. So far, no consensus has been reached on the primary/basal function of melanins or the nature of their formation as either a primary or a side effect. On the contrary, it is a matter of constant discussion and dispute.

Melanins are difficult to define due to their structural complexity, but in lichens, they mostly belong to either the eumelanins or allomelanins^14,15^. In lichens, melanins may be synthesised in some parts of the thallus, e.g., in the cilia, cortex layer, or throughout the whole thallus^14^. Interestingly, the production of melanins could be environmentally induced by, for example, UV-B radiation, as was evidenced in *Lobaria pulmonaria*^8,16^ and observed in many species growing in sun-exposed habitats^17^.

In lichens exposed to high solar radiation, melanins are accumulated in the upper cortex, which protects the algal cells against UV^18^. Photosynthetically active radiation (PAR) also causes harmful effects on lichens since it leads to heat stress, a faster rate of drying after hydration, oxidative stress and photoinhibition^14^. However, lichens developed several mechanisms to protect against photoinhibition, including synthesising cortical pigments^8^. McEvoy et al*.*^16^ found that in *Lobaria pulmonaria* exposed to intense light, melanins play a photoprotective role, and melanised thalli displayed a minor reduction in the maximum quantum yield of PSII photochemistry. Similar protective roles of melanin against excessive solar radiation in lichens were observed in *Umbilicaria decussata*^19^, and *Bryoria fuscescens*^20^ Melanins also play important roles in responding to other abiotic factors since they are involved in desiccation and osmotic stress tolerance^21^, heavy-metal tolerance^22^, X-ray protection^23^ and represent strong antioxidants^24^.

Another potential role of melanins in lichens concerns the protection of the thalli against temperature stress. To date, such a phenomenon has been observed in several studies on free-living fungi. In detail, the melanisation of *Cryptococcus neoformans* led to an increase in its tolerance to heat and cold stress^25^, while conidia of melanin-deficient mutants of *Monilinia fruticola* were more sensitive to high temperatures^26^. Similarly, the melanised conidia of *Cordyceps* spp. proved to be more tolerant to high temperatures than non-melanised ones^27^. However, such a mode of action of melanins is poorly understood and probably relates to their capacity to reduce stress-induced ROS production^22^. On the other hand, strong melanisation can increase thallus temperature in lichens^16^, which could induce heat stress. Since melanins are very effective in absorbing solar radiation and dissipating it in the form of heat, the potential side-effect of increased thallus temperature cannot be omitted. In cold polar regions, where thallus temperatures are often not optimal for photosynthesis, melanin-induced warming can be an advantage because heated thalli melt snow allows hydration and physiological activity at temperatures below 0 °C^28,29^. On the other hand, in the temperate climate zone, thallus melanisation should provide a balance between protection against UV and the side effects of heat stress.

It is widely believed that heat stress is one of the most severe threats to living organisms due to its direct effect on metabolism. Extreme climatic phenomena such as heat waves, heavy rainfall and prolonged droughts are one of the main problems associated with ongoing climate change. Summer heatwaves are projected to increase in amplitude and frequency in the near future^30,31^, but the consequences of such extreme events on lichens are largely unknown. Lichens can tolerate harsh environmental conditions when dry and metabolically inactive but are very sensitive when metabolically active^32^; thus, heat stress seems particularly dangerous for hydrated lichens. Consequently, the occurrence of extreme rainfall with simultaneous high air temperatures should be considered a particularly adverse phenomenon. Many studies have reported a global increase in extreme rainfalls associated with rising temperatures, both in historical records and future climate scenarios^33^. Even in the near future (2020–2049), heat waves are predicted to be almost twice as frequent as compared to the modelled historical period in Central Europe^34^. At the same time, most climate models also indicate an increase in the frequency and intensity of extreme rainfall, mainly in terrestrial areas of medium and high latitudes^35^. Increasingly, frequent extreme events also affect the soil and associated organisms, such as lichens, as the soil temperature is related to various environmental factors, e.g. air temperature near the surface, amount of energy received from the Sun or surface albedo. Several studies confirmed an increasing trend in soil temperatures, mainly during the spring and summer, especially in the upper soil layer^36,37^.

The study aimed to determine the impact of heat stress on both symbiotic partners’ physiology and whether the lichen thallus’s strong melanisation could be an additional adaptive feature to endure high temperatures in an increasingly common climate with heavy rainfall and high-temperature combination. Specifically, we aimed to verify whether strongly melanised thalli of *Cetraria aculeata* are more resistant to high-temperature stress than poorly melanised thalli, thus determining the relevance of melanins in response to short-term heat stress in a metabolically active state. We set the following hypotheses: (1) Melanised thalli would be more effective in defending against oxidative stress induced by heat stress; (2) The photosynthesis of the algal partner would be the most sensitive element in the heat stress response. We believe that our study will provide valuable knowledge to understand further the adaptation of lichens to heat stress and their response mechanisms.

## Results

### Melanin UV–Vis spectrum

**Figure 1:**
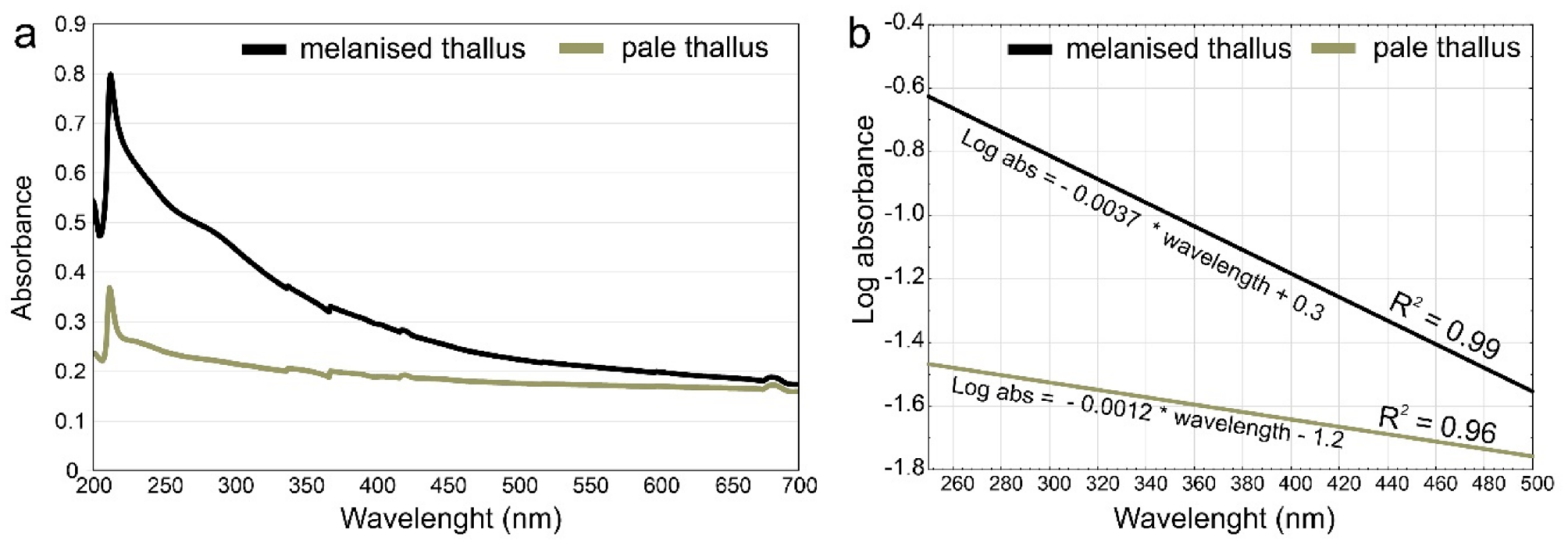
The UV–VIS spectra of pure melanin extracted from melanised and pale thalli of *Cetraria aculeata* (**a**) and the linear curves obtained after plotting the logarithm of absorbance against the wavelength (**b**). The formula of the function and the coefficient of determination (R^2^) are given.

The UV–Vis spectra of the extracted and purified pigment from melanised and pale thalli of *C. aculeata* are shown in Fig. 1. The absorption profile of melanised and pale thalli was very similar. The absorbance increases progressively towards shorter wavelengths. The maximum absorption value of the spectrum of melanin extracted from both melanised and pale thalli was observed at 216 nm in the ultraviolet region but decreased progressively as the wavelength increased toward the visible region (Fig. 1a). The spectra of extracted melanin did not show any other absorption peaks between 260 and 280 nm regions, which indicates that melanin does not contain proteins and nucleic acids^38^. After plotting the logarithm of absorbance against the wavelength, negative slopes were recorded for melanin extracted from both melanised (− 0.0037) and pale (− 0.0012) lichen thalli (Fig. 1b). This indicates that the black pigments are melanins since such slopes of linear plots are often used as an essential criterion for the identification of melanins and have been previously obtained in certain terrestrial fungi (see^39^ and reference herein). Melanin extracted from melanised thalli showed higher values (2.33 ± 0.77; n = 3) of A_300_/A_600_ ratios, related to the oxidation state and the range size of melanin molecules, than that from pale thalli (1.28 ± 0.16; n = 3). This may suggest that the pigments extracted from melanised thalli are a more complex mixture of melanin molecules than that from pale thalli, with variability in size and degree of oxidation.

The extraction and purification processes afforded 5.7 ± 2.8 mg and 0.7 ± 0.2 mg of pigment from 4 g DW of melanised and pale lichen thalli, respectively.

### Thallus temperature and relative water content

**Figure 2:**
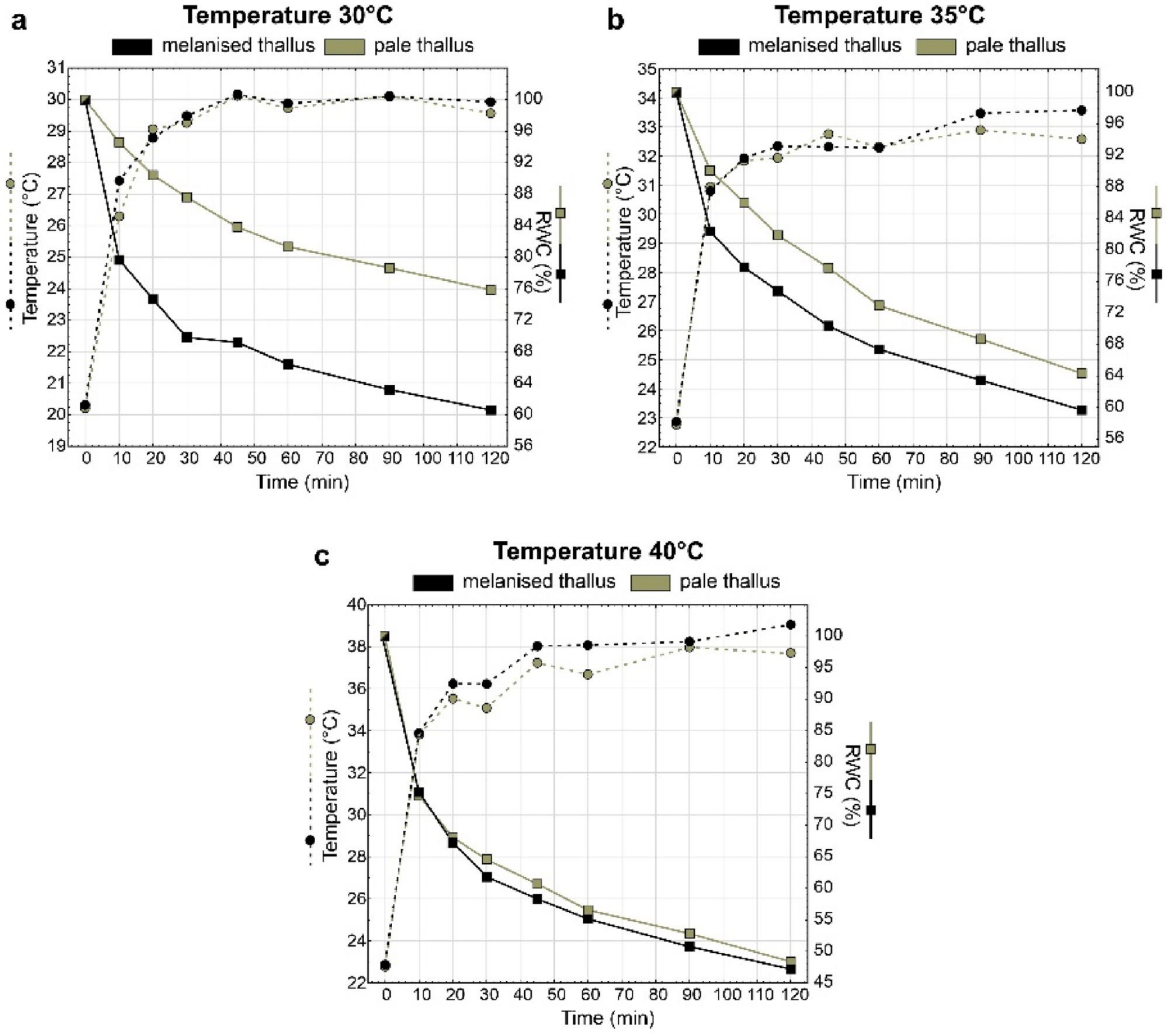
Changes in thallus surface temperature (mean; n = 6) and relative water content (RWC) of melanised and pale thalli of *Cetraria aculeata* during 2-h heat stress experiment at 30 °C (**a**), 35 °C (**b**) and 40 °C (**c**).

The lichen thallus temperature during heat stress at 30 °C of both melanised and pale thalli was characterised by a similar course during the 2-hour experiment, reaching maximum temperature after 45 min (Fig. 2a). As regards temperature 35 °C, both types of thalli also showed similar temperature variations; melanised thalli reached a slightly higher temperature than pale thalli after 90 and 120 min; however, the differences were not significant (Fig. 2b, Table S1). During heat stress with a temperature of 40 °C, melanised thalli had a higher temperature than pale thalli in most time intervals, and these differences were significant at 30, 45, 60 and 120 min (Fig. 2c; Table S1). In the case of heat stress at 35 °C and 40 °C, the temperature of the thalli did not exceed the set temperature (Fig. 2b,c). The most significant differences in relative water content of melanised and pale thalli were observed during heat stress with a temperature of 30 °C. The differences between thallus types decreased at 35 °C, while at 40 °C the changes of RWC were at a similar level in both types of the thallus (Fig. 2). As a rule, melanised thalli reached lower RWC compared to pale thalli at each temperature and time interval (Fig. 2).

### Integrity of cell membranes

**Figure 3:**
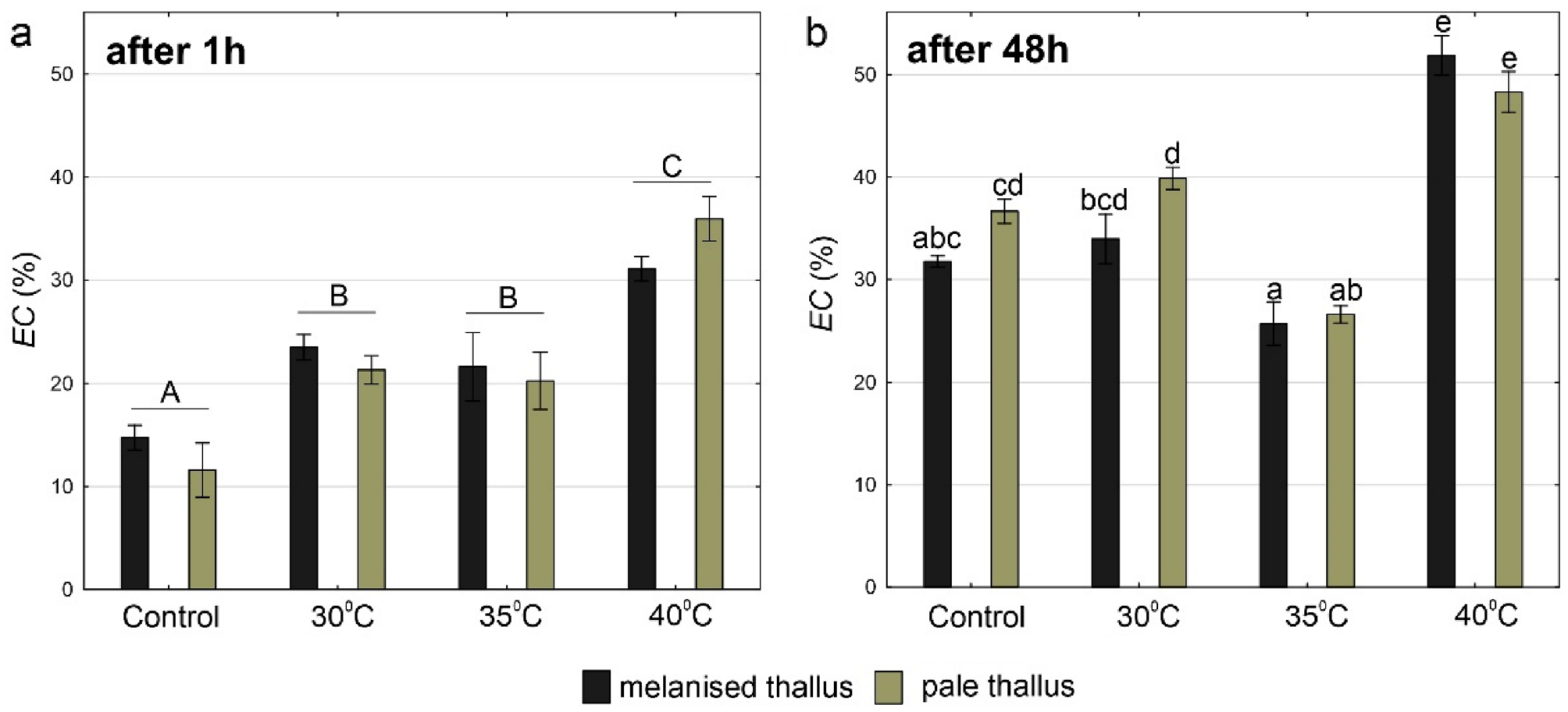
The *EC* parameter in melanised and pale thalli of *Cetraria aculeata* (means ± SE; n = 6) 1 h (**a**) and 48 h (**b**) after heat treatment at different temperatures. The different letters above the bars indicate statistically significant differences (p < 0.05). Lowercase letters indicate a statistically significant interaction between temperature and thallus type; capital letters indicate a significant effect of temperature. For details on the main effects and interactions, see Table S2.

As regards cell membrane integrity, the significant effect of temperature after 1 h and significant interaction between temperature and thallus type after 48 h have been recorded (Fig. 3a,b, Table S2). After 1 h, the lowest EC values (up to 15%) were observed in the control group, which differed significantly from the remaining experimental groups. Significantly higher EC was recorded after treatment with a temperature of 40 °C (Fig. 3a). After 48 h, significantly higher EC values (exceeding 50%) were observed in both melanised and pale thalli after treatment with a temperature of 40 °C. At lower temperatures, cell membrane damage was lower in melanised thalli than in the pale thalli; however, the differences were not significant (Fig. 3b).

On analysing the effect of heat stress after 48 h, regeneration was not observed in any case. A significant increase in *EC* after 48 h compared to 1 h was observed at 30 °C and 40 °C in both melanised and pale thalli (Student’s t-tests, p < 0.05; Fig. S1).

### Membrane lipid peroxidation

**Figure 4:**
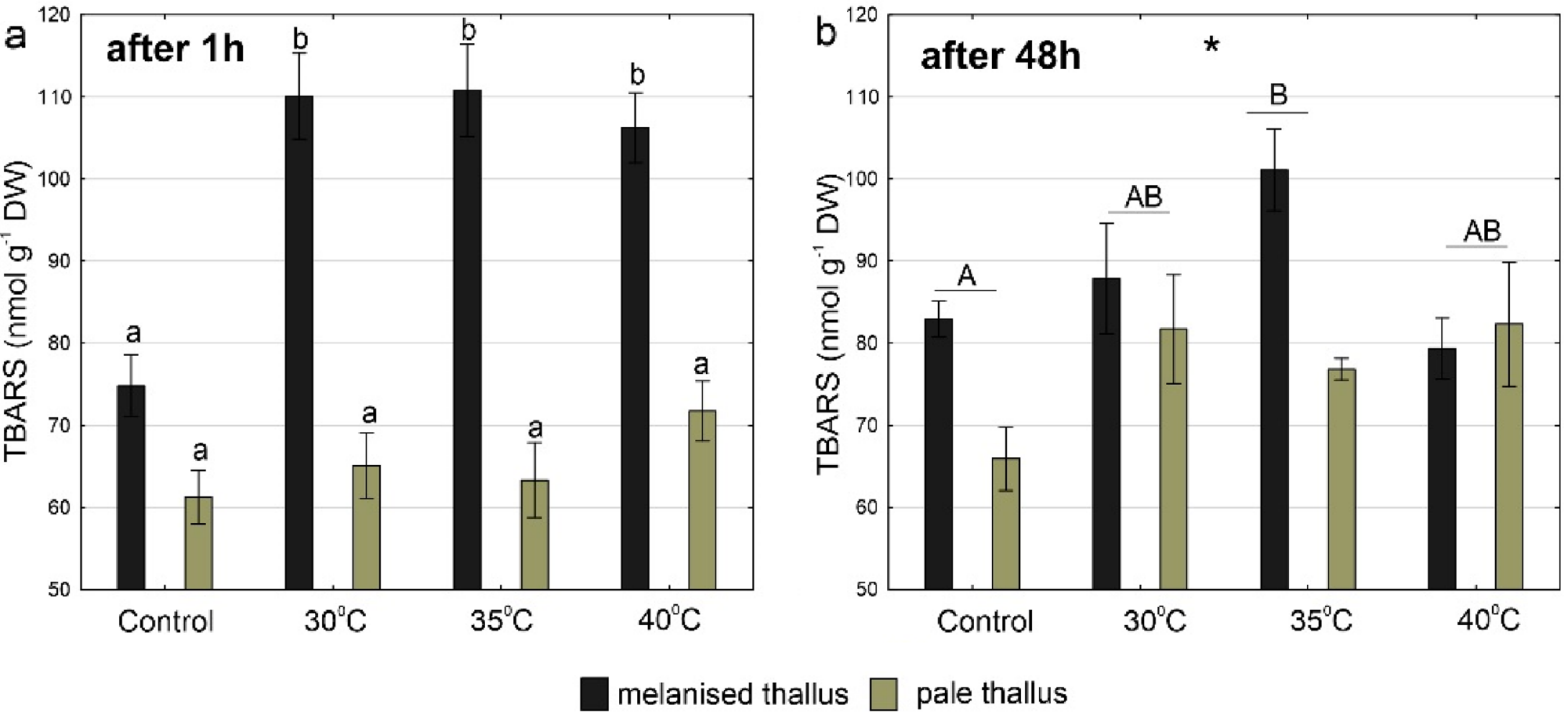
TBARS concentrations in melanised and pale thalli of *Cetraria aculeata* (means ± SE; n = 6) 1 h (**a**) and 48 h (**b**) after heat treatment at different temperatures. The different letters above the bars indicate statistically significant differences (p < 0.05). Lowercase letters indicate a statistically significant interaction between temperature and thallus type; capital letters indicate a significant effect of temperature. The asterisk indicates a statistically significant effect of thallus type. For details on the main effects and interactions, see Table S2.

Regarding TBARS concentrations in *C. aculeata*, a significant interaction between temperature and thallus type after 1 h and significant main effects of temperature and thallus type after 48 h were observed (Fig. 4a,b, Table S2). After 1 h, the lowest TBARS concentrations were recorded in the control group and pale thalli at each temperature and differed significantly from TBARS concentrations in melanised thalli at 30 °C, 35 °C and 40 °C, which were nearly twice as high, reaching above 110 nmol g^-1^ DW (Fig. 4a). TBARS levels were still lower in pale thalli compared to melanised thalli after 48 h. The highest TBARS concentrations (ca. 100 nmol g^−1^ DW) were observed at 35 °C, which differed significantly from the control group (Fig. 4b).

Lower TBARS concentrations were observed in melanised thalli after 48 h compared to 1 h after heat stress; this decrease was significant at 30 °C and 40 °C (Fig. S2a). On the contrary, pale thalli regeneration after 48 h was not observed. Moreover, a significant increase in TBARS concentrations after 48 h at 35 °C was recorded (Fig. S2b).

### Dehydrogenase activity

**Figure 5:**
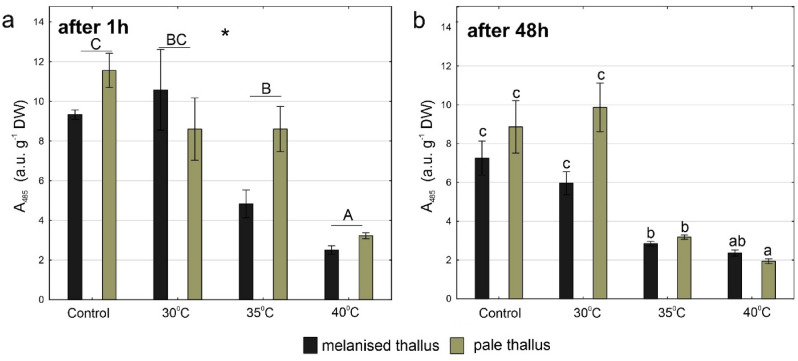
Dehydrogenase activity expressed as absorbance at 485 nm on g DW in melanised and pale thalli of *Cetraria aculeata* (means ± SE; n = 6) 1 h (**a**) and 48 h (**b**) after heat treatment at different temperatures. The different letters above the bars indicate statistically significant differences (p < 0.05). Lowercase letters indicate a statistically significant interaction between temperature and thallus type; capital letters indicate the significant effect of temperature. The asterisk indicates a statistically significant effect of thallus type. For details on the main effects and interactions, see Table S2.

Dehydrogenase activity in *C. aculeata* was influenced by temperature and thallus type after 1 h and the interaction of these two factors after 48 h (Fig. 5a,b, Table S2). After 1 h, the highest dehydrogenase activity was observed in control and 30 °C groups, while significantly lowest values were recorded at 40 °C in both thallus types (Fig. 5a). Generally, dehydrogenase activity was higher in pale thalli compared to melanised thalli. After 48 h, dehydrogenase activity decreased with the increasing temperature reaching significantly the lowest values at 35 °C and 40 °C in both melanised and pale thalli (Fig. 5b).

After 48 h from heat stress, both melanised and pale thalli dehydrogenase activity decreased in most groups (Fig. S3a,b). In detail, a significant decrease after 48 h compared to 1 h was observed at 35 °C in melanised thalli (Fig. S3a) and at 35 °C and 40 °C in pale thalli (Fig. S3b).

### Maximum quantum yield of PSII photochemistry and JIP-test parameters

**Figure 6:**
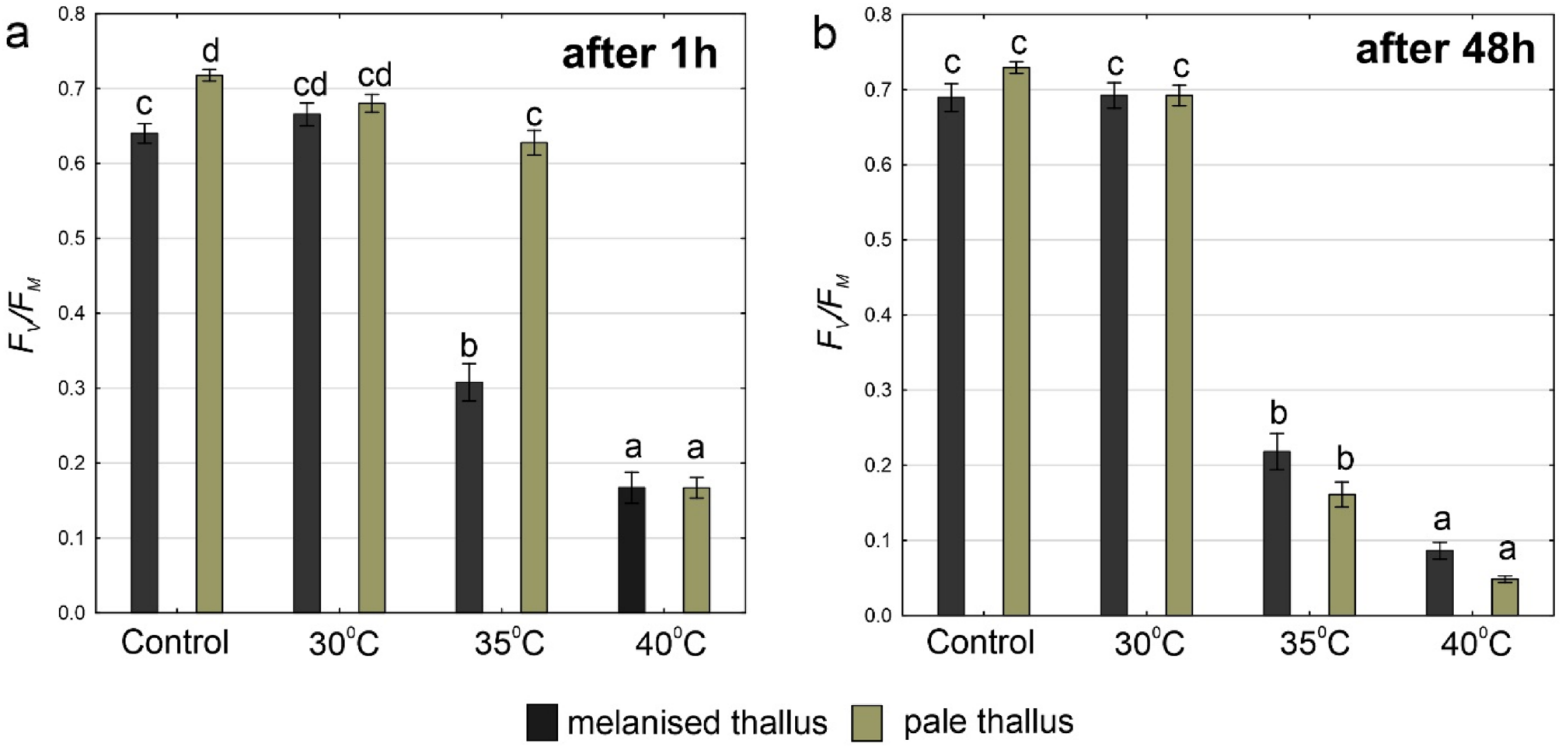
The *F*_*V*_*/F*_*M*_ parameter in melanised and pale thalli of *Cetraria aculeata* (means ± SE; n = 10) 1 h (**a**) and 48 h (**b**) after heat treatment at different temperatures. The different letters above the bars indicate statistically significant differences (p < 0.05). Lowercase letters indicate a statistically significant interaction between temperature and thallus type. For details on the main effects and interactions, see Table S2.

As regards the *F*_*V*_*/F*_*M*_ parameter, significant interactions between temperature and thallus type after 1 h and 48 h were recorded (Fig. 6a,b, Table S2). After 1 h, the highest *F*_*V*_*/F*_*M*_ values were observed in both melanised and pale thalli in control, at 30 °C, and in pale thalli at 35 °C. In contrast, a significant decrease was observed in melanised thalli at 35 °C and in both thallus types at 40 °C (Fig. 6a). After 48 h, the *F*_*V*_*/F*_*M*_ was at the high level typical of healthy lichens (ca. 0.7) in control and 30 °C groups in both thallus types, while significantly decreased at higher temperatures reaching the lowest values at 40 °C. After heat stress at 35 °C and 40 °C, the *F*_*V*_*/F*_*M*_ was lower in pale thalli than the melanised thalli; however, the differences were not significant (Fig. 6b).

Analysing the effect of heat stress on *F*_*V*_*/F*_*M*_ after 48 h compared to 1 h, the increase was not observed in either melanised or pale thalli. A significant decrease in *F*_*V*_*/F*_*M*_ parameter after 48 h compared to 1 h was observed at 35 °C and 40 °C in both melanised and pale thalli (Fig. S4a,b).

Heat stress also influenced other parameters characterising PSII functionality. After 1 h, a decrease in Area, T*F*_*M*_, PI_ABS_, Psi(E_0_), Phi(E_0_), Phi(P_0_), ET_0_/RC and an increase of *F*_*0*_, ABS/RC and DI_0_/RC parameters were recorded. These trends were evident at 35 °C and 40 °C in melanised thalli and at 40 °C in pale thalli. Generally, after 48 h, these trends deepened in both thallus types (Fig. S5).

## Discussion

Regarding the effect of heat stress on lichen physiological condition, many studies found that elevated temperature negatively affects the cover, diversity and photosynthetic capacity of lichens in drylands^40^, polar regions^41,42^ and heath communities^43^. Most of them examined the effect of slightly elevated temperatures, e.g. 1 °C during different lengths of exposure periods. The results of warming studies of various durations (i.e. from 6 weeks to max. 5 years) showed an inconsistent effect on lichens; some indicated a negative effect^44^, while others positive^45^. However, as regards long-term warming studies^41^ (e.g. 16 years), most results demonstrated mainly adverse effects on lichen communities. It is widely known that temperature increases will directly affect photosynthesis and respiration. In metabolically active wet lichens, the lethal temperatures for photosynthesis in photobionts usually range between 30 to 44 °C^46,47^. However, the resistance to heat stress certainly depends on lichen species. For example, in lichens from the arid region of South Africa, the lethal temperature ranged between 44.1 and 48.1 °C^48^. In contrast, Marin et al*.*^42^ reported that photosystem II activity persisted only up to 30 °C in four Antarctic lichen species. Our results showed that for photosynthetic apparatus in *C. aculeata,* the critical temperature is around 35 °C and that melanised thalli were more sensitive to heat stress. Nevertheless, it should be remembered that the desiccated inactive state in lichens protects them against heat-induced destruction, and several lichen species are considered resistant to high-temperature stress with heat resistance ranging from 70 to 101 °C for air-dried thalli^46^. The high rate of water loss from the thallus in the natural environment^49^ and consequent evaporative cooling protects lichens against heat-induced damage. We also noted that a decrease in relative water content (RWC) allowed the temperature of the thallus to be kept at a constant level, slightly lower than the ambient temperature (Fig. 2). However, the predicted extremes in weather with heavy rainfall events during hot summer season associated with global warming could result in the exposure of hydrated lichens to extreme temperatures that exceed their tolerance thresholds.

Most of the research on the effect of high temperature on lichen vitality focuses solely on the photosynthesis process of the photobiont. Contrarily, there are no detailed studies on the influence of heat stress on the physiological condition of the mycobiont. Generally, most fungi need warm conditions for hyphae growth; however, increasing temperature leads to disturbances in their life cycle and cellular processes^50^. Although fungal species differ in optimum growth temperature, most are mesophiles that grow in the temperature range of 5–35 °C, with optima between 20 and 25 °C^51^. Nevertheless, there is limited information about the physiological response of lichenized fungi to heat stress. Since lichens are symbiotic organisms with obligatory dependence, adapting them to survive under heat stress is crucial. Our research showed that the condition of the mycobiont is also considerably reduced due to heat stress. This was manifested by a significant increase in the level of damage to cell membranes, increased membrane lipid peroxidation and decreased dehydrogenase activity.

Damage to lichen thalli from heat stress can occur in natural habitats due to intensive sun exposure after heavy rainfall. Heat injuries were observed a long time ago, where the damaged forms of *Cladonia* species were found in wet places of the bog, where the water temperature was considerably increased^52^. Lichens are poikilohydrous organisms, and their internal water content is quickly adjusted to the water potential of the surrounding environment. An increase in temperature usually means a simultaneous increase in water loss due to an increased deficit in air saturation with water, which initially decreases the thallus temperature through evaporative cooling. It is the most important mechanism that allows lichens to survive heat stress due to their ability to dry quickly and intensively after sun exposure because, in the air-dried state, most lichens are highly tolerant to heat^47^. The obtained results are extremely important in the context of the lack of a mechanism increasing tolerance to heat stress in lichens, well developed in vascular plants, which is probably related to the fact that wet thalli are not commonly exposed to heat stress in natural conditions^47^. Nevertheless, given the intensification of extreme weather phenomena, the impact of heat stress on the physiological condition of lichens should be seriously considered, given our results, which showed that even short-term exposure to heat stress in a hydrated state could lead to significant damage. Soil surface temperature under full radiation conditions can exceed 60 °C^53^. Moreover, lichen thalli in a dry state can heat up to much higher temperatures than the air temperature. Lange^54^ demonstrated that the thalli of *Cladonia furcata* heat up to a temperature above 50 °C in conditions of intense sun radiation at an air temperature of 26.2 °C. On the contrary, its thalli in contact with the soil surface warmed up to even 69.6 °C. Consequently, encountering such conditions after heavy rainfall can severely affect lichen vitality.

Exploring the detrimental and beneficial effects of lichen melanisation, it is essential to note that pigmentation has been recognised as an ancient adaptation mechanism for extracting thermal energy from radiation^55^. It is a basic attribute in living organisms that absorb radiation energy and regulate temperature. Darker pigments absorb more radiation than lighter ones and transfer more heat that may provide an adaptive advantage at higher latitudes, but becomes unfavourable in warmer climates due to the risk of overheating^56,57^. Thermal melanism is a phenomenon known in various organisms since the last century^58^. The lightness of the colours is also crucial for the thermoregulation of fungi^59^. For lichens living in cold polar regions, thallus temperatures are often not optimal for photosynthesis; thus, melanin-induced warming can be an advantage because heated thalli melt snow allowing hydration and physiological activity at temperatures below 0 °C^28,29^. Conversely, it can be expected that in the temperate climate zone, mycobiont melanisation should constitute a trade-off between protection against UV and avoidance of damage caused by high temperatures. On the other hand, there are also reports on certain pigments that increase thermotolerance. For example, melanised *Cryptococcus neoformans* cells had a higher survival rate after heat or cold shock than non-melanised cells^25^, suggesting a role for melanin in protection against heat and cold stress. However, Kershaw^29^ observed in a laboratory experiment that dark and pale lichen thalli differed in temperature by 6–7 °C, indicating that strongly melanised thalli are additionally exposed to the adverse effects of heat stress. Our results did not confirm our initial hypothesis of the role of melanin in protection against heat stress because exposure to high temperatures caused deterioration of all tested parameters of the fungal partner. We observed that highly melanised thalli are generally more sensitive to heat stress compared to pale ones. In particular, this concerns the maximum quantum yield of PSII photochemistry and the level of membrane lipid peroxidation. Given that this effect is not related to higher thalli temperatures, as may be the case in the environment due to stronger solar heating of dark thalli, it can be concluded that extreme summer temperatures in temperate climates may lead to significant physiological deterioration of melanised thalli. Nevertheless, we observed an interesting phenomenon related to the change in the level of membrane lipid peroxidation 48 h after heat stress in melanised thalli. Although TBARS concentrations were generally higher in melanised thalli than in pale thalli, they significantly decreased after 48 h. It may indicate a reduction in the level of oxidative stress over time and may be related to the activation of defence mechanisms as a result of heat stress. One reason for this phenomenon may be the antioxidant activity of melanin, as TBARS content increased over time in the pale thalli. Melanins were shown to display a high antioxidant activity in many fungal species^11,22^. Their high concentration in the thallus may favour the protection of cells against oxidative damage as they may act as scavengers of ROS^60^.

McEvoy et al*.*^16^ suggested that the exposure to elevated thallus temperatures of tripartite old forest lichen *Lobaria pulmonaria* may be a critical factor for the survival of melanic thalli. However, in the natural environment, this species is not exposed to high solar radiation; therefore, the light-shielding effect of melanins is probably more beneficial than the adverse heat-induced stress, at least in natural forest habitats^16^. Contrarily, *Cetraria aculeata* grows in various biomes worldwide, including dry and hot grassland and steppe habitats^61^. Therefore, the consequences of heat stress for *C. aculeata* will undoubtedly be more considerable than for *L. pulmonaria*. Many organisms on Earth rely on different pigments to optimise energy exchange between them and their environment, thereby determining their geographical distribution and adaptation to changes in climate^56^.

**Figure 7:**
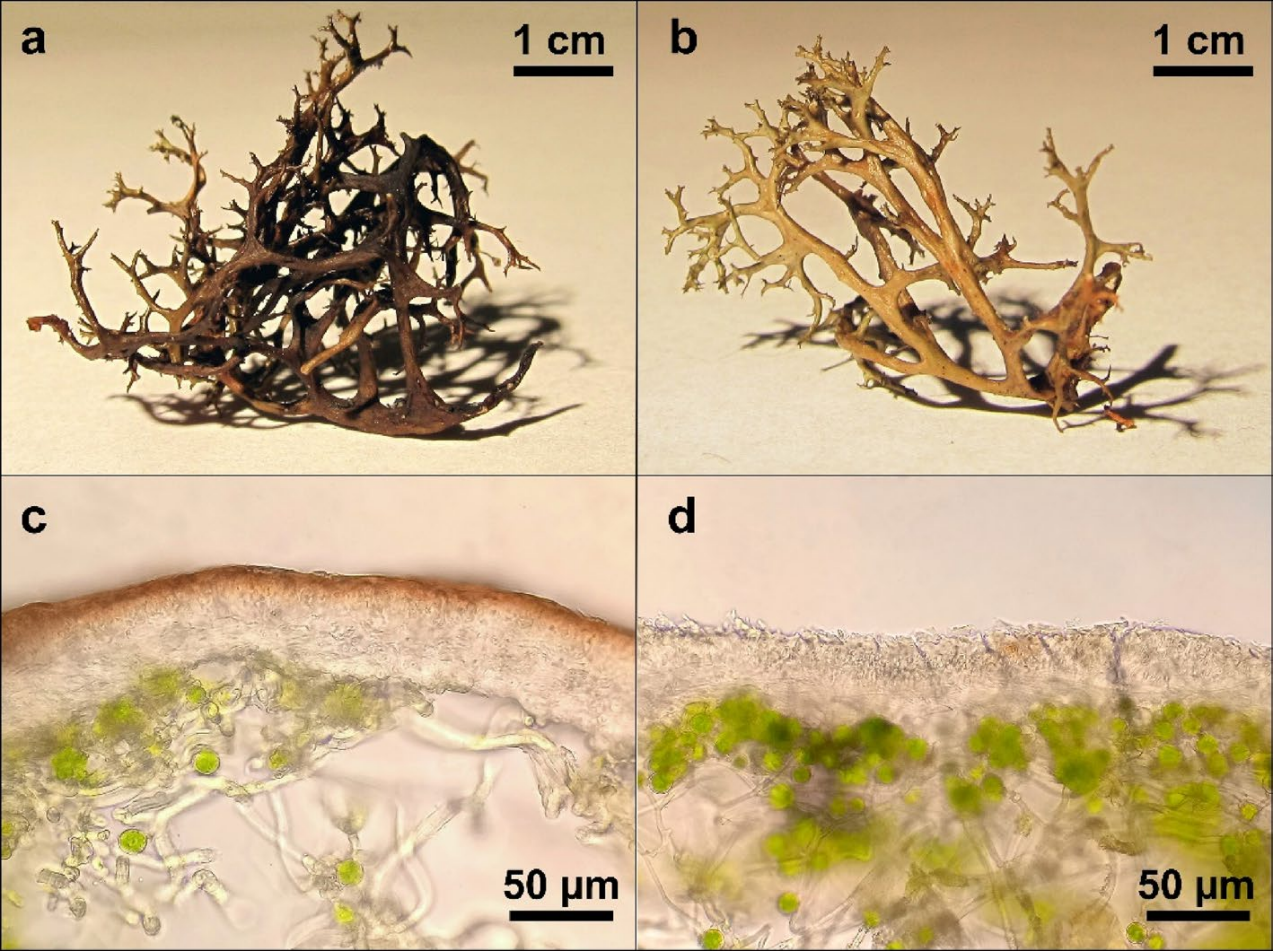
The thalli of *Cetraria aculeata* intended for experiments*—*melanised (**a**,**c**) and pale (**b**,**d**). Whole thalli (**a**,**b**) and thallus cross-sections (**c**,**d**) showing highly pigmented upper cortex layer of melanised thallus (**c**).

We observed an interesting phenomenon of faster water loss from melanised thalli compared to pale ones, which may indicate that dark thalli are better adapted to protect against heat stress by more rapid water evaporation, which in turn, protects them from heat-induced damage. Determining the cause of this phenomenon would require further research. Nevertheless, we suppose that it may be related to the different structures of the thallus in melanised and pale individuals of *C. aculeata* lichens. Melanised lichens had a considerably lower density of hyphae in the medulla compared to pale ones (see Fig. 7). It is well-known that water relations proved to be influenced by specific morphological and anatomical characters of the thallus^49,62^. The structure of the medulla proved to be an essential factor affecting water relations in lichens. The thalli with loosely-woven arachnoid medulla show more rapid water exchange with the air and have a relatively low saturation capacity, whereas the dense packing of hyphae in the medulla determines the higher water retention capacity and ability to retain water for a comparatively long period^63^. Similarly, Phinney et al*.*^17^ reported that the rate of water loss was higher and the duration of metabolically active periods lower in melanic lichen species compared to those containing usnic acid. Nevertheless, in our case, it is not related to the influence of solar radiation, which induces faster heating of dark thalli.

In conclusion, our study showed that the critical temperature for metabolism in hydrated *C. aculeata* thalli is around 35 °C. Both symbiotic partners responded to heat stress, manifested by the decreased maximum quantum yield of PSII photochemistry in the algal partner and high level of cell membrane damage, increased membrane lipid peroxidation and reduced dehydrogenase activity. We found that highly melanised thalli are generally more sensitive to heat stress than pale ones, excluding the role of melanins as compounds protecting against heat stress. Therefore, in the temperate climate zone, mycobiont melanisation imposes a trade-off between protection against UV and avoidance of damage caused by high temperatures. Therefore, maintaining the thallus in good physiological condition requires high plasticity in producing melanin pigments. Since the observed effect in our experiment was not associated with a higher temperature of the thallus, as may occur in the natural environment due to stronger heating of dark thalli by solar radiation, it can be concluded that extreme temperatures in the environment during the summer in a temperate climate may lead to a significant deterioration of the physiological condition of melanised thalli. Nevertheless, we observed some aspects that may support highly melanised thalli to combat heat stress. This was evidenced by the reduction in the level of membrane lipid peroxidation after time, which may suggest greater efficiency of defence mechanisms and indicate the antioxidant properties of melanins. Secondly, melanised thalli can lose water faster than pale ones, which may indicate that dark thalli are better adapted to protect against heat stress by more rapid evaporation of water. Since recent reports showed that in just a decade, global warming has severely increased the frequency of heat and rainfall extremes, and new heat and precipitation record is expected in the coming decade^64^, many lichen species may require a great deal of plasticity to maintain their physiological state at a level that ensures their survival.

## Methods

### Selected species

*Cetraria aculeata* (Schreber) Fr. is a fruticose epigeic lichen with coarsely dichotomously branched thalli, forming irregular, shrubby tufts often of brown or almost black colour at sun-exposed sites^65^. The photosynthetic partner represents algae belonging to *Trebouxia jamesii*^66^. It is a cosmopolitan species occurring most frequently in open polar and boreal environments from the maritime Antarctic to the high Arctic and in high mountains in wind-exposed situations as well as dry and hot grassland and steppe habitats in temperate climate zone^67^. The species is characterised by high phenotypic plasticity and can form substantial modification of thallus morphology and anatomy that represent one of the most extreme cases of infraspecific phenotypic variation found in lichens^61^. The species has a three-layered, thick cortex layer and a dense, brown outer layer with an accumulation of dead cells^61^, which provides an excellent protective barrier against excessive solar radiation. So far, the presence of melanins in this species has not been proven.

### Sample collection and handling

Lichen samples were collected in the September of 2022 from semi-natural sandy grassland in Klucze village (Pustynia Błędowska desert, S Poland; 50°20′28.0"N; 19°32′36.2"E). The climate of the sampling area is classified as temperate oceanic with warm summer (Cfb) according to the updated Köppen-Geiger climate classification^68^. The most critical climatic features are presented in the climatic diagram prepared for the years 1986–2022 based on records from the local meteorological station (Fig. S6; IMGW station code: 250190390, raw data were obtained from the Institute of Meteorology and Water Management—National Research Institute, Poland).

Melanised and pale thalli were collected from fully sun-exposed and partly shaded sites, respectively (Fig. 7). The study area covers initial psammophilous grassland communities of Spergulo-Corynephoretum adjacent to the young pine forest belonging to Cladonio-Pinetum^69^. The collected materials were packed in paper envelopes and transported to the lab. Then the thalli were thoroughly cleaned of soil debris, bryophytes and plants using tweezers and a brush.

### Melanin extraction and UV–Vis analysis

Melanised (n = 3) and pale (n = 3) lichen thalli were used to confirm the presence of melanins. The extraction procedure followed that of Rassabina et al*.*^15^ with minor modifications. Lichen thalli were dried at 40 °C for 10 min. 4 g DW of lichen thalli were homogenised in laboratory mill A11 (IKA, Poland) into a fine powder. Then the powder was placed into a test tube with 45 ml of 2 M NaOH (10.5 pH). After incubation for 24 h on a vibration shaker (350 rpm, 25 °C), the mixture was filtered and centrifuged (MPW-352R, Poland) at 10,000 × *g* for 10 min to remove fungal hyphae and algal cells. The supernatant was acidified by adding 5 M HCl to pH 2.5 to precipitate melanin, incubated for 12 h at 20 °C, and centrifuged again at 10,000 × *g* for 10 min. The supernatant was discarded, and the crude melanin was further shaken in concentrated HCl solution (37%) for 15 min to hydrolyse melanin-bound proteins, carbohydrates and lipids. After centrifugation, the pellet was washed five times with distilled water to remove acid and successively washed with chloroform, ethyl acetate, and three times with acetone to wash away secondary metabolites, lipids and other residues, and dried in a heater at 40 °C (SML-30/250, Poland). Finally, the purified melanin was the remaining dark-brown powder without foreign inclusions. The melanin powders were weighed on an analytical balance Quintix 125D-1CEU (Sartorius, Germany) to determine their content in melanised and pale thalli. The absorption spectra of melanin alkaline solution (25 μg/ml in 0.1 M NaOH) were recorded in the UV/visible spectrum (200–700 nm) in quartz cuvettes with an optical path of 1 cm on a spectrophotometer Jasco V-650 (Japan). The A_300_/A_600_ ratios were calculated. They give information on the oxidation state and the range size of melanin molecules. Melanin oxidation induces lower absorbance values at 600 nm (A_600_), and the A_300_/A_600_ absorbance ratio was proposed as a measure of the oxidation extent; high values corresponded to greater oxidized melanin molecules^70^.

### Experimental design

**Figure 8:**
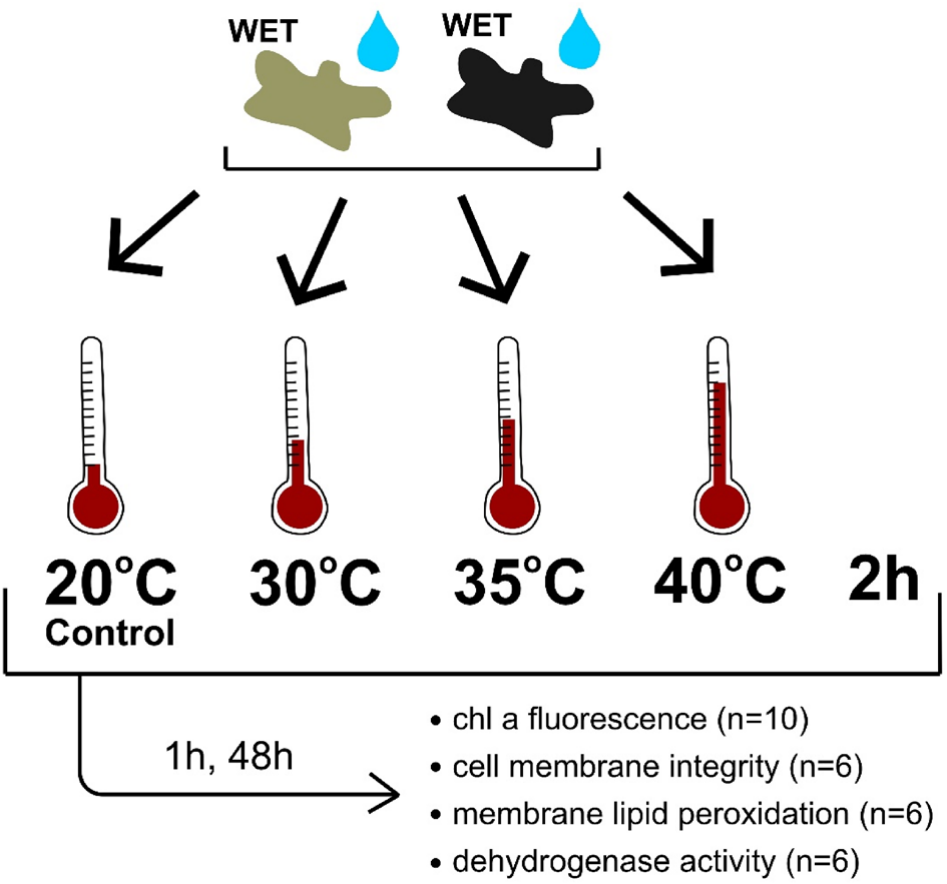
Diagram showing the design of the experiment. The number of replicates intended for particular analyses for a given experimental group is provided.

Prior to the experiments, lichen thalli were hydrated and kept for 48 h in a chamber with 95% relative humidity and temperature of 20 °C at low light intensity (ca 10 μmol photons m^−2^ s^−1^) to reactivate physiological activity and maintain membrane integrity of cells^71^. The material was divided into two groups constituting melanised and pale lichen thalli. A total of 8 experimental groups were considered (Fig. 8). The thalli (both melanised and pale) were exposed to temperatures ranging from 30 °C to 40 °C in a metabolically active state (fully hydrated thalli). The thalli were exposed to 30 °C, 35 °C and 40 °C for 2 h. Wet thalli were put in a heater at low light intensity (ca 10 μmol photons m^−2^ s^−1^) on petri dishes with wetted filter paper under the thalli to maintain an active metabolic state in the thalli during the experiment. The control groups of melanised and pale thalli were placed in a humid chamber at 20 °C at low light intensity (ca 10 μmol photons m^−2^ s^−1^). The maximum temperature (40 °C) was chosen since similar air temperatures during the summer in temperate climates during heat waves are frequently observed. After exposure to heat stress, the thalli were hydrated and placed in a climatic chamber (ca. 95% relative humidity, 20 °C), and measurements were taken after 1 h and 48 h. The single replicate for each of the subsequent analyses was a single individual/fragment of thallus from a single individual, so each replicate constituted an independent sample.

The experimental conditions have been established to reflect best the conditions that may occur in the environment during hot summer when metabolically active lichen thallus encounters high-temperature conditions excluding the additional influence of solar radiation. Larson^72^ provided evidence that a species with a similar growth pattern to the species examined here, i.e. *Cladonia stellaris*, loses water from fully hydrated thalli relatively slowly, reaching 25% relative water content (RWC) after 2 h (experiment conducted under conditions reflecting natural environmental conditions). Barták et al*.*^73^ showed that most lichen species show no limitations of maximum PSII quantum yield (*F*_*V*_*/F*_*M*_) in the thalli desiccating from a wet state (RWC: 100%) to a semi-dry state (RWC: approx. 35%), and only RWCs below 25% causes significant decline in the *F*_*V*_*/F*_*M*_. Moreover, in the related species *Cetraria islandica* during the initial phase of water loss from the thallus up to RWC = 60%, the quantum yield of PS II and *F*_*V*_*/F*_*M*_ even increased or remained at a constant level^74^. Furthermore, the minimum duration of the metabolically active period tested in dark (melanic) lichen species forming hemispherical cushions was found to be 131 min, starting from fully saturated thalli under 350 µmol photons m^−2^ s^−1^ light conditions^17^. In accordance with these previous reports, we set the duration of the experiment at 2 h for fully hydrated thalli, eliminating the effect of radiation and wind to determine only the influence of temperature while assuming that the thalli will be exposed to a given temperature in a metabolically active state during this time.

### Thallus temperature and relative water content measurements

The temperature and relative water content (RWC) in lichen thalli were monitored throughout 2-hour experiments at 10, 20, 30, 45, 60, 90 and 120 min after placing lichens in the heater. The temperature was measured with IR 110-6S Infrared Thermometer (Voltcraft^®^, Germany). Six temperature measurements were taken in different parts of the thalli in each time interval and then averaged. The emissivity was set to 0.957 based on the average emissivity reported for the plants^75^. The technique was non-invasive and measured infrared (IR) radiation emitted from the thalli. The infrared sensor detected the emitted heat radiation and converted this information into a temperature value.

The relative water content (RWC) in the thallus was evaluated by following the procedure of Jonsson et al. with minor modifications^76^. Samples were weighed on precision balance (Kern PCB 250–3, Germany). RWC (%) was calculated according to the formula:

$$RWC=\left(FW-DW_{des}\right)/\left(FW_{sat} -DW_{des}\right)\times 100\left[\%\right]$$

where: RWC is the current relative water content in the thallus at a specific time interval, FW is the current fresh weight of the thallus, and DW_des_ is the dry weight of the thallus determined after drying at 60 °C over silica gel in a heater for 12 h, FW_sat_ is the fresh weight of the thallus when fully hydrated, determined by soaking lichen in water for 3 h at 20 °C and removal of the excess water by a quick shaking of the thallus prior to the weighing.

### Integrity of cell membranes

Ca 150 mg of lichen material was weighed and soaked in 50 ml of distilled water in glass weighting bottles, covered with glass stoppers, and shaken on a vibrating shaker for 1 h (150 rpm; Vibramax 100, Heidolph Instruments, Germany). The initial electrical conductivity (*Ci*) of the distilled water was measured using a conductivity meter (Seven Go Duo SG23-FK5, Mettler Toledo, Switzerland). After that, the conductivity of the samples was measured after soaking the thalli (*Cv*), and the samples were boiled for 10 min at 100 °C to disrupt cell membranes. The samples were then cooled to room temperature, and the conductivity was measured again (*Cf*). Finally, the relative electrical conductivity (*EC*) considered as the level of loss of membrane integrity was calculated according to the formula: ((*Cv* − *Ci*)/*Cf*) × 100 (%). Six replicates were measured for each experimental group.

### Assessment of membrane lipid peroxidation

The level of membrane lipid peroxidation in lichen samples was estimated using the thiobarbituric acid-reactive substances (TBARS) assay following the procedure of Heath and Packer^77^ with modifications of Politycka^78^. First, ca 40 mg of air-dried lichen material were weighed. The samples were homogenised in a porcelain mortar using 1.5 ml of ice-cold 0.25% (w/v) thiobarbituric acid (TBA) in 10% trichloroacetic acid (TCA). These mixtures were then heated in a water bath (JWE 357, Elpin-Plus, Poland) at 95 °C for 30 min. After that, the samples were cooled to room temperature and were centrifuged at 12,000 × *g* for 15 min (Centrifuge 5424, Eppendorf, Poland). The absorbance of the supernatant was measured at 532 nm (Genesys 180 UV–Vis spectrophotometer, Thermo Fisher Scientific, USA) and corrected for nonspecific absorption at 600 nm. The extinction coefficient specific for thiobarbituric acid-malondialdehyde complex (TBA-MDA; 155 mM^−1^cm^−1^) was used for the calculation of the concentration of lipid peroxidation products (TBARS). The level of membrane lipid peroxidation was expressed as nmol of TBARS per gram of DW of lichen thalli. Six replicates were measured for each experimental group.

### Dehydrogenase activity

The vitality of the mycobiont, which represents ca. 90% of the lichen biomass, was verified by the reduction of 2,3,5-triphenyltetrazolium chloride (TTC) to red-coloured triphenylformazan (TPF), which is directly linked to dehydrogenase activity and represents the activity of the mitochondrial respiratory chain^79^. The application of tetrazolium salt is a widely used method to measure redox reactions in plant and fungal cells^80,81^. TTC successfully competes with NAD + for electrons, and thus constitutes an artificial electron acceptor. The TTC accepts electrons directly from the low potential cofactors of the NADH dehydrogenase (complex I) in the respiratory chain^82^. As TTC accepts electrons, it is reduced to a red formazan. The colorimetric method based on TTC reduction provides an accurate assay of dehydrogenase activity. The lichen material (ca. 40 mg) was incubated in the dark for 20 h at 25 °C in 2 ml of 0.6% TTC (Sigma-Aldrich) and 0.005% Triton X 100 solution (Sigma-Aldrich) in 50 mM sodium phosphate buffer adjusted to pH 6.8. Then the solutions were removed, and samples were rinsed in distilled water until complete removal of Triton X. The samples were then dried on filter membrane. Water-insoluble formazan was extracted with 4 ml of ethanol (95%, Sigma-Aldrich) at 65 °C for 1 h. The test tubes were then centrifuged at 4000 × *g* for 10 min and the supernatant absorbance was measured at 485 nm. The results were expressed as absorbance units on a dry weight of the thalli. Three pseudoreplicates per single lichen sample were measured. Six true replicates for each experimental group were analysed.

### Chlorophyll fluorescence measurements

Chlorophyll fluorescence was measured using Handy-PEA + fluorimeter (Plant Efficiency Analyzer, Hansatech Instruments Ltd, United Kingdom). Lichen samples were dark-adapted with clips for 20 min and then illuminated for 1 s with 650-nm light-emitting LED diodes of 3000 µmol m^−2^ s^−1^. Ten replicates for each experimental group were measured.

The physiological stress of the lichen photobiont was assessed by classical indicator of the maximum quantum yield of PSII photochemistry *F*_*V*_*/F*_*M*_ = (*F*_*M*_ − *F*_*0*_)/*F*_*M*_, where *F*_*M*_ is a maximum and* F*_*0*_ is a minimum chl *a* fluorescence and *F*_*V*_ = (*F*_*M*_ − *F*_*0*_) variable fluorescence. The *F*_*V*_*/F*_*M*_ greater than 0.63 were considered to correspond to physiologically healthy thallus^83^. Other chlorophyll fluorescence parameters that were analysed are presented in Table S3.

### Data analysis

Absorbance readings from the UV–Vis spectra of melanin pigment alkaline solution in the range from 250 to 500 nm (reading resolution of 1 nm) were averaged over three analysed samples for melanised and pale lichen thalli. Then, the variables were logarithmised, and the relationships between the logarithm of absorbance and the wavelength were tested with linear regression analysis to determine the function formulas and the coefficient of determination (R^2^) separately for melanised and pale lichen thalli.

For statistical analyses, the following factors were considered: temperature (20 °C = control, 30 °C, 35 °C, 40 °C), thallus type (pale, melanised) and time after heat stress (1 h, 48 h). Following Levene’s test, used to assess the equality of variances, Student’s t-tests (p < 0.05) were run to test the significance of differences in thallus temperature between melanised and pale thallus at 10, 20, 30, 45, 60, 90, 120 min after placing lichens in the heater exposed to heat stress at 30 °C, 35 °C and 40 °C. Two-way analyses of variance (two-way ANOVA; p < 0.05) were performed to assess the effect of thallus type and temperature on *EC*, TBARS concentration, dehydrogenase activity, and *F*_*V*_*/F*_*M*_ 1 h and 48 h after heat stress. The significance of differences between particular experimental groups was then verified with Tukey's HSD post-hoc tests (p < 0.05). To assess the possibility of recovery or deterioration of physiological condition after heat stress over time, the Student’s t-tests (p < 0.05) were applied to test the significance of differences in parameters above between 1 and 48 h after heat stress for pale and melanised lichen thalli, separately. Before performing these analyses, the normality distribution within groups was checked using the Kolmogorov–Smirnov test. The Brown–Forsythe tests were used to verify the equality of variances. The box-cox transformation was applied when necessary. Statistical analyses were performed using STATISTICA 13 (TIBCO Software Inc., Palo Alto, CA, USA).

The spider plots were created to visualise the effect of heat stress on the parameters characterising PSII functionality after 1 h and 48 h in the pale and melanised thalli of *C. aculeata*. Graphs were used to check which parameter responded most strongly to heat stress. The plots were based on the values normalised to the control, which made it possible to compare the parameters measured on the various scales.

## Supplementary Information

Supplementary Information.

## Data Availability

All data generated or analysed during this study are included in this published article (and its Supplementary Information files).
