# Supplementary material for: Effect of thallus melanisation on the sensitivity of lichens to heat stress
Source: Sci Rep. 2023 Mar 28;13:5083. doi: 10.1038/s41598-023-32215-1 (PMC10049980; doi:10.1038/s41598-023-32215-1)
Supplement: Supplementary file 1 — Supplementary Information. [file 41598_2023_32215_MOESM1_ESM.pdf]

Supporting information (electronic supplementary materials available on-line)

Effect of thallus melanisation on the sensitivity of lichens to heat stress

Karolina Chowaniec[1,2], Ewa Latkowska[3] & Kaja Skubała[1]*

[1] Institute of Botany, Faculty of Biology, Jagiellonian University, Gronostajowa 3, 30-387 Kraków, Poland
[2] Doctoral School of Exact and Natural Sciences, Jagiellonian University in Kraków, prof. S. Łojasiewicza 11, 30-348 Kraków, Poland
[3] Laboratory of Metabolomics, Faculty of Biochemistry, Biophysics and Biotechnology, Jagiellonian University, Gronostajowa 7, 30-387, Krakow, Poland
* Corresponding author. E-mail address: kaja.skubala@uj.edu.pl (K. Skubała)

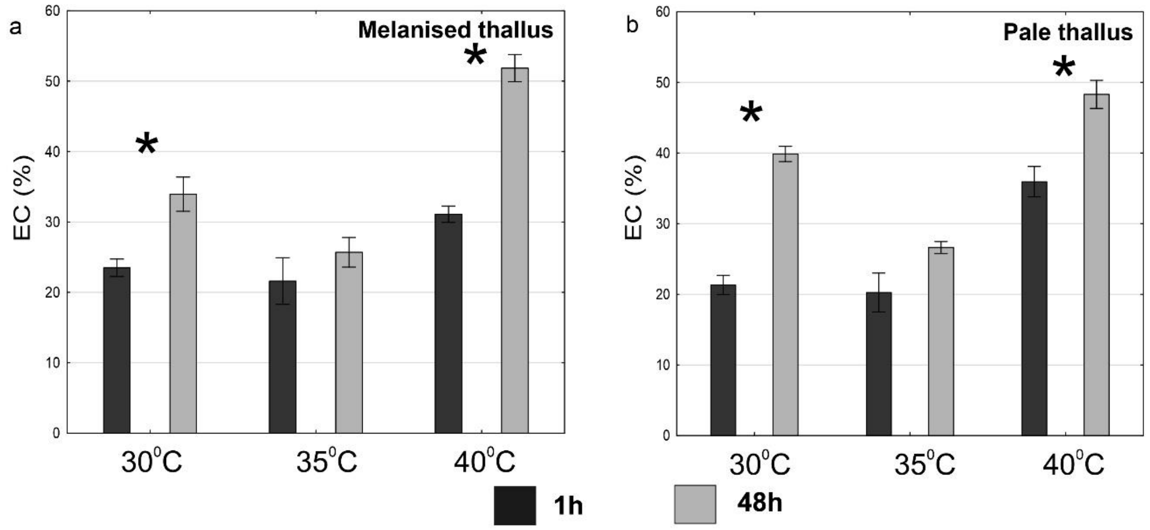

Figure S1. The *EC* parameter in melanised (**a**) and pale (**b**) thalli of *Cetraria aculeata* (means ± SE; n = 6) 1h and 48h after heat treatment at different temperatures. The asterisks indicate significant differences between 1h and 48h after heat stress within the same temperature according to the Student's t-tests ($p < 0.05$).

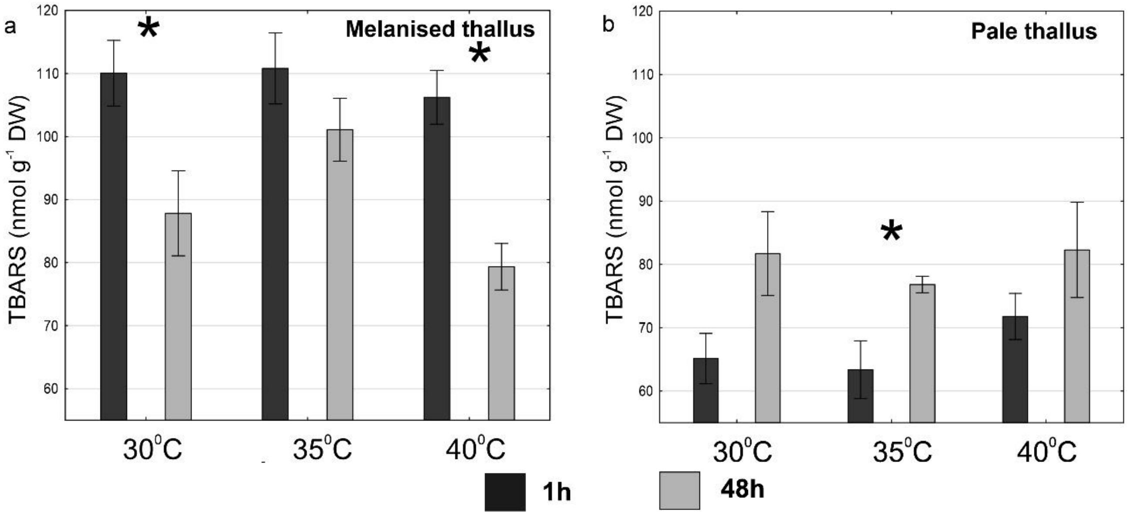

Figure S2. TBARS concentrations in melanised (**a**) and pale (**b**) thalli of *Cetraria aculeata* (means ± SE; n = 6) 1h and 48h after heat treatment at different temperatures. The asterisks indicate significant differences between 1h and 48h after heat stress within the same temperature according to the Student's t-tests ($p < 0.05$).

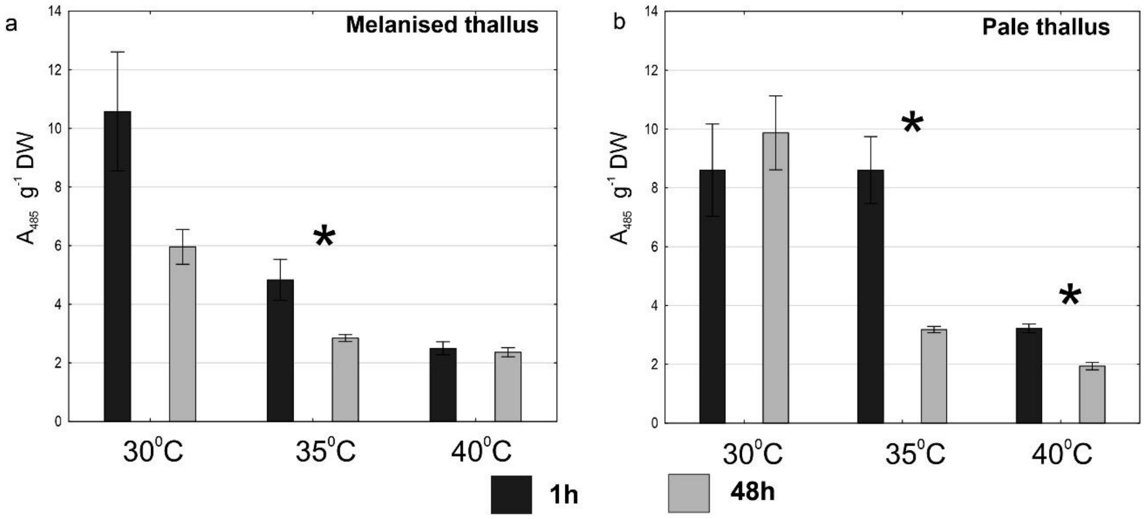

Figure S3. Dehydrogenase activity expressed as absorbance at 485 nm on g DW in melanised (**a**) and pale (**b**) thalli of *Cetraria aculeata* (means ± SE; n = 6) 1h and 48h after heat treatment at different temperatures. The asterisks indicate significant differences between 1h and 48h after heat stress within the same temperature according to the Student's t-tests ($p < 0.05$).

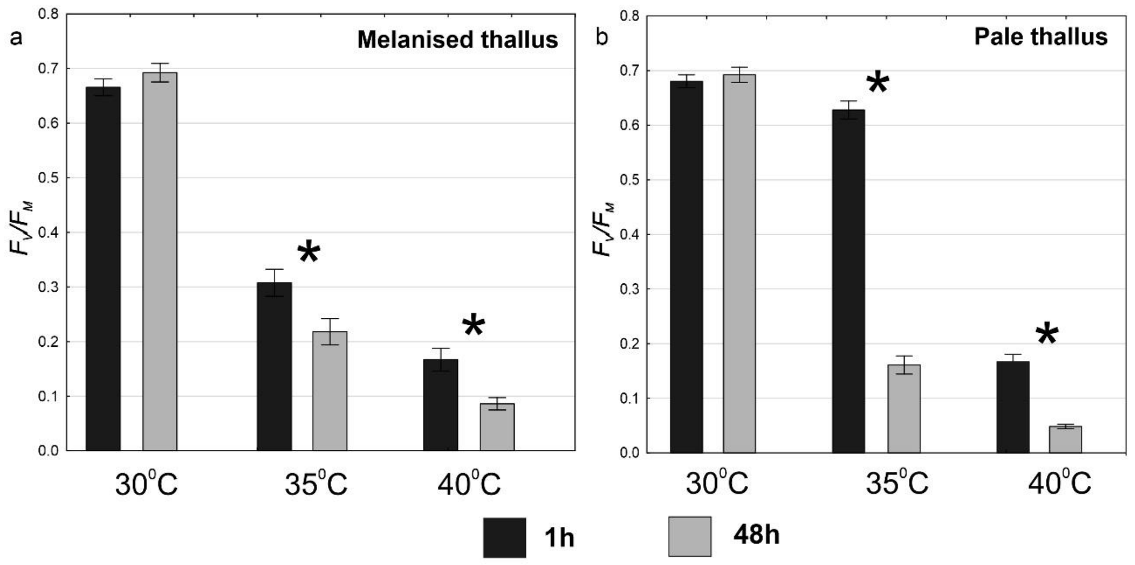

Figure S4. The F_V/F_M parameter (means ± SE; n = 10) in melanised (**a**) and pale (**b**) thalli of *Cetraria aculeata* 1h and 48h after heat treatment at different temperatures. The asterisks indicate significant differences between 1h and 48h after heat stress within the same temperature according to the Student's t-tests ($p < 0.05$).

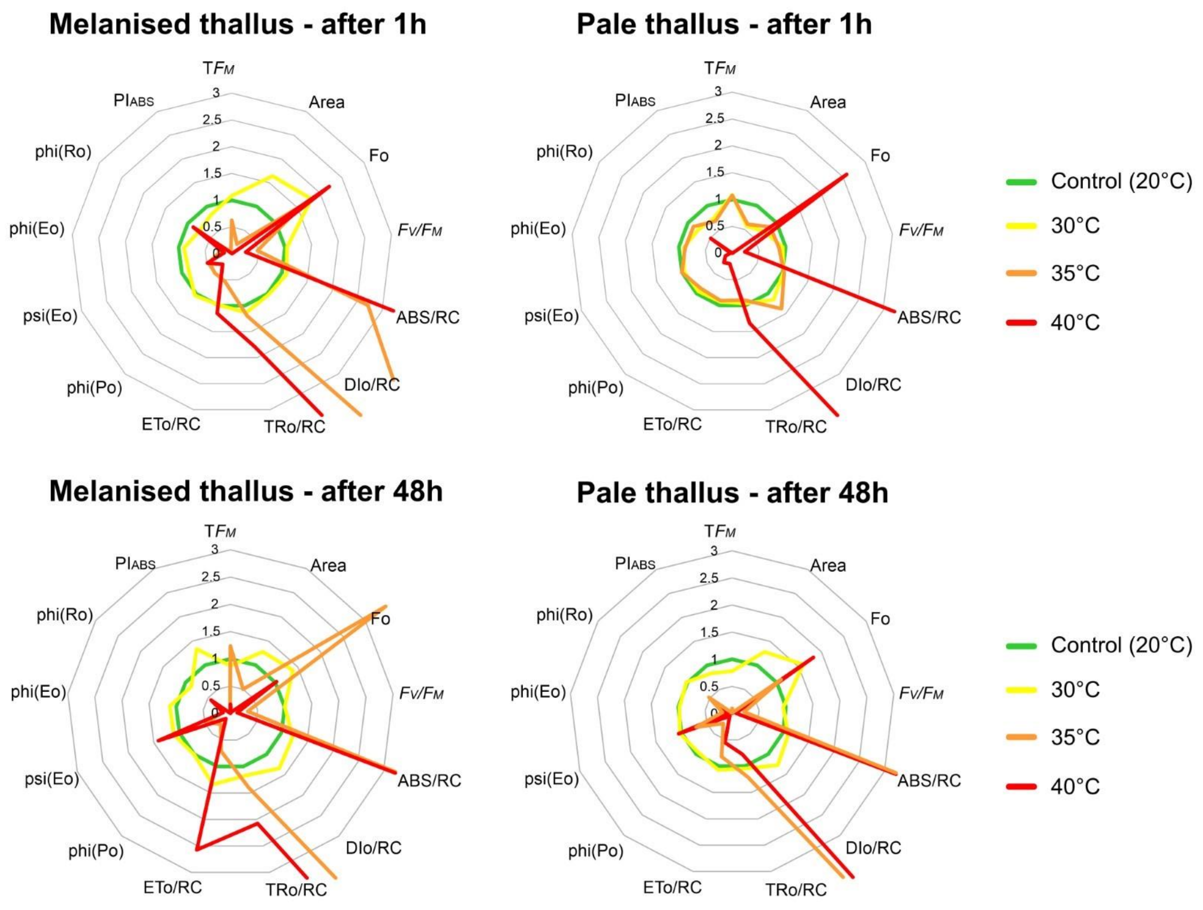

Figure S5. The spider plots showing the effect of temperature on various photosynthetic parameters characterising PSII functionality 1h and 48 h after heat treatment of melanised and pale thalli of *Cetraria aculeata*. The plots are based on normalized values to control treatment, enabling comparison of the variables measured on different scales. A detailed description of all parameters is provided in Table S2.

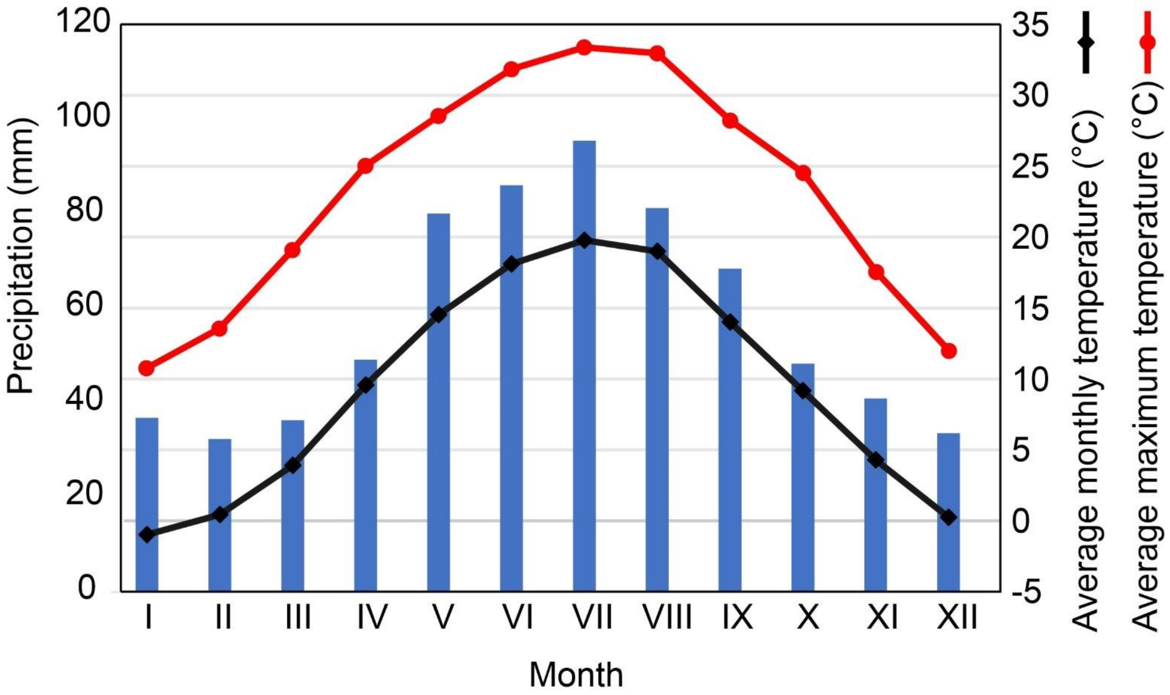

Figure S6. The climatic diagram for average monthly temperature (black line), average maximum temperature (red line) and precipitation (blue bars) for meteorological station in the study area (IMGW station code: 250190390) calculated for the years 1986-2022.

Table S1. Temperatures of pale and melanised thallus (means ± SD) throughout two hours of experiments at 10, 20, 30, 45, 60, 90, 120 minutes after placing lichens in the heater along with the results of Student's t-tests ($p < 0.05$) testing the significance of differences between pale and melanised thalli for each time point, separately.

Temperature (°C)	Time (min)	Temperature pale thallus (°C; mean ± SD)	Temperature melanised thallus (°C; mean ± SD)	t	p
30	0	20.2±0.1	20.3±0.2	-0.89	0.393
	10	26.3±1.6	27.4±0.9	-1.51	0.162
	20	29.1±1.0	28.8±0.8	0.53	0.607
	30	29.3±1.0	29.5±0.5	-0.46	0.655
	45	30.1±1.3	30.2±0.8	-0.08	0.936
	60	29.7±0.2	29.9±0.5	-0.65	0.533
	90	30.1±0.6	30.1±0.4	0.06	0.954
	120	29.6±0.6	29.9±0.5	-1.19	0.260
35	0	22.8±0.6	22.9±0.7	-0.27	0.796
	10	30.9±0.9	30.8±1.0	0.27	0.789
	20	31.8±0.7	31.9±1.0	-0.17	0.871
	30	31.9±0.9	32.3±0.7	-0.87	0.405
	45	32.8±1.8	32.3±0.6	0.56	0.586
	60	32.3±0.8	32.3±0.7	0.11	0.915
	90	32.9±1.0	33.5±1.1	-0.93	0.375
	120	32.6±0.8	33.6±1.2	-1.72	0.117
40	0	22.8±0.6	22.9±0.7	-0.27	0.796
	10	33.8±1.3	33.8±1.1	-0.02	0.981
	20	35.5±0.8	36.2±1.1	-1.31	0.220
	30	**35.1±0.8**	**36.2±0.9**	**-2.36**	**0.040**
	45	**37.2±0.7**	**38.0±0.4**	**-2.42**	**0.036**
	60	**36.7±1.2**	**38.1±1.0**	**-2.23**	**0.049**
	90	38.0±0.7	38.1±1.4	-0.21	0.838
	120	**37.7±1.3**	**39.1±0.8**	**-2.25**	**0.048**

Table S2. The results of two-way analysis of variance ($p < 0.05$) for the effect of temperature and thallus type on *EC* parameter, TBARS concentrations, dehydrogenase activity, and F_V/F_M parameter for *Cetraria aculeata* 1h and 48h after heat treatment.

Parameter	Time after heat stress	Independent variables	F	p	df	Error df	R^2
EC	1h	Temperature	31.04	<0.001	3	40	0.71
		Thallus type	0.09	0.763	1		
		Temperature × Thallus type	1.44	0.246	3		
	48h	Temperature	73.50	0.000	3	40	0.85
		Thallus type	3.10	0.086	1		
		Temperature × Thallus type	3.46	0.025	3		
**Parameter**	**Time after heat stress**	**Independent variables**	**F**	**p**	**df**	**Error df**	R^2
TBARS concentration	1h	Temperature	10.44	<0.001	3	40	0.82
		Thallus type	130.11	<0.001	1		
		Temperature × Thallus type	6.29	0.001	3		
	48h	Temperature	2.97	0.043	3	40	0.40
		Thallus type	9.56	0.004	1		
		Temperature × Thallus type	2.78	0.053	3		
**Parameter**	**Time after heat stress**	**Independent variables**	**F**	**p**	**df**	**Error df**	R^2
Dehydrogenase activity	1h	Temperature	34.32	<0.001	3	40	0.89
		Thallus type	4.83	0.034	1		
		Temperature × Thallus type	2.20	0.103	3		
	48h	Temperature	105.79	<0.001	3	40	0.89
		Thallus type	1.25	0.269	1		
		Temperature × Thallus type	4.30	0.010	3		
**Parameter**	**Time after heat stress**	**Independent variables**	**F**	**p**	**df**	**Error df**	R^2
F_V/F_M	1h	Temperature	437.57	<0.001	3	72	0.95
		Thallus type	80.19	<0.001	1		
		Temperature × Thallus type	41.66	<0.001	3		
	48h	Temperature	951.25	<0.001	3	72	0.98
		Thallus type	1.59	0.211	1		
		Temperature × Thallus type	3.92	0.012	3		

Table S3. The selected chlorophyll fluorescence (OJIP) derived parameters calculated on the basis of fast fluorescence kinetics used in further analyses.

Fluorescence parameter	Description
Basic	
F_0	Minimal fluorescence intensity
F_V/F_M	Maximum quantum yield of PSII photochemistry
TF_M	Time to reach maximum fluorescence (F_M)
Area	Density area over the ChF transient delimited by a horizontal line at F_M
The specific energy fluxes per reaction centre (RC)	
ABS/RC	Specific absorption flux per reaction centre
DI_0/RC	Dissipated energy flux per reaction centre
TR_0/RC	Trapped energy flux per reaction centre
ET_0/RC	Electron transport flux per reaction centre
Quantum yields and efficiencies	
Phi (P_0)	Probability that an absorbed photon will be trapped by the reaction centre of PSII
Psi (E_0)	Probability that a trapped exciton moves an electron into the electron transport chain beyond QA
Phi (E_0)	Quantum yield of electron transport
Phi (R_0)	Quantum yield of reduction of end electron acceptors at the PS I acceptor side
Performance index	
PI_{ABS}	Performance index (potential) for energy conservation from photons absorbed by PSII to the reduction of intersystem electron acceptors
